# Adjuvant chemotherapy non‐adherence, patient‐centered communication, and patient‐level factors in elderly breast and colon cancer patients

**DOI:** 10.1002/cam4.5884

**Published:** 2023-05-06

**Authors:** Kerri‐Anne R. Mitchell, Joseph R. Boyle, Lenka Juricekova, Richard F. Brown

**Affiliations:** ^1^ Public Health Program Austin College Sherman Texas USA; ^2^ Department of Biostatistics Virginia Commonwealth University School of Medicine Richmond Virginia USA; ^3^ Department of Health Behavior and Policy Virginia Commonwealth University School of Medicine Richmond Virginia USA

**Keywords:** adherence, oncology, patient‐centered care, patient‐provider communication

## Abstract

**Background:**

We examined patient‐level factors (patient characteristics, disease and treatment factors, and patient experience), patient‐centered communication (PCCM), and non‐adherence to adjuvant chemotherapy (AC) guidelines among breast and colon cancer patients to inform AC adherence promotion and improve clinical outcomes.

**Methods:**

Descriptive statistics for patient‐level factors, PCCM, and AC non‐adherence (primary non‐adherence, non‐persistence at 3 and 6 months) were obtained. Multiple logistic regression models were used to estimate AC non‐adherence after accounting for the identified patient‐level factors.

**Results:**

The majority of the sample (*n* = 577) were White (87%), breast cancer patients (87%), and reported PCCM (provider communication score ≥ 90%, 73%, provider communication score = 100%, 58%). All three levels of AC nonadherence were significantly higher in breast cancer patients (69%, 81%, and 89% for primary non‐adherence, and non‐persistence at 3 and 6 months, respectively) than colon cancer patients (43%, 46%, and 62%, respectively). Male sex, survey assistance, and low/average ratings of a personal doctor, specialist, and healthcare were associated with lower PCCM. Older age, breast cancer diagnosis, and diagnosis group following 2007–2009 increased the likelihood of all three levels of AC non‐adherence. Comorbidities and PCCM‐90 were exclusively associated with non‐persistence at 3 months.

**Conclusions:**

Adjuvant chemotherapy non‐adherence varied by cancer diagnosis and treatment factors. The relationship between PCCM and AC non‐adherence differed by level of PCCM, time period, and the presence of comorbidities. AC guideline adherence, communication, and value‐concordant treatment should be assessed and compared simultaneously to improve our understanding of their interrelationships.

## 1 INTRODUCTION

Breast and colon cancers are among the most prevalent cancer sites in the United States.^1^ Almost 2 million Americans were diagnosed with either breast or colon cancer between 2016 and 2020 and nearly half a million died (includes rectal cancer deaths as colon and rectal cancer deaths are not reported separately).^2^, ^3^, ^4^, ^5^, ^6^ Surgery followed by chemotherapy, also known as adjuvant chemotherapy (AC), significantly improves clinical outcomes such as survival and recurrence in these patients^7^, ^8^ and is recommended for patients with localized or locoregional disease. Yet, there is wide variation in AC adherence guidelines and persistence with treatment regimens among breast (10%–83.7%)^9^, ^10^ and colon (36%–38%) cancer patient groups.^11^, ^12^ Primary adherence is receiving any amount of treatment as recommended,^13^, ^14^ and is distinct from persistence, which reflects the proportion of time that a patient complies with a treatment regimen as recommended.^13^, ^14^, ^15^ Improvements in primary non‐adherence and lack of persistence among older patients with breast and colon cancer diagnoses would support improved clinical outcomes.

### 1.1 Factors associated with treatment non‐adherence

Factors that inhibit adherence to AC guidelines in cancer patients are not fully understood but include: (a) patient characteristics (e.g., age,^11^, ^12^, ^16^, ^17^, ^18^ sex,^11^, ^12^ race, and ethnicity,^11^, ^12^, ^16^, ^19^, ^20^ marital status,^16^ socioeconomic status,^11^, ^17^, ^21^ Medicare insurance,^11^ and patient choice^18^), and (b) disease factors (e.g., symptom experience/co‐morbidities,^18^, ^19^ metastatic disease^18^) and treatment factors (e.g., treatment modality,^22^ non‐academic center care,^11^ and time of diagnosis^11^, ^16^, ^17^). While several correlates reflect clinical factors, non‐clinical factors are also implicated, and linked to the patient care experience, most notably patient‐provider communication and the therapeutic relationship.^23^, ^24^ The literature suggests that optimal patient‐centered communication (PCCM) improves the navigation of challenging clinical topics and strengthens patient‐provider relationships by promoting patient engagement, trust, and understanding.^25^, ^26^, ^27^ PCCM has been shown to improve treatment adherence in the primary care setting,^28^ and for various illnesses including diabetes^29^ and HIV^30^; however, more research is needed to understand the nature of this relationship among eligible breast and colon cancer patients and identify multiple targets for intervention especially in sub‐groups with high rates of AC non‐adherence.

To address this knowledge gap, we proposed three aims and three hypotheses: Aim 1—Obtain and compare the rates of non‐adherence at three levels (primary non‐adherence, non‐persistence at 3 months, and non‐persistence at 6 months) among breast and colon cancer patients, Hypothesis 1—The rates of primary non‐adherence and non‐persistence to AC guidelines at 3 and 6 months will vary by cancer site, Aim 2—Explore associations between PCCM and patient‐level factors (patient characteristics, disease and treatment characteristics and patient experience indicators), primary non‐adherence, and non‐persistence to AC guidelines at 3 and 6 months among elderly breast and colon cancer patients, Hypothesis 2—The associations between PCCM and the identified patient‐level factors will vary by level of AC non‐adherence (primary non‐adherence and non‐persistence to AC guidelines at 3 and 6 months), Aim 3—Evaluate whether PCCM (a high provider communication rating) is associated with lower AC primary non‐adherence and non‐persistence at 3 and 6 months among breast and colon cancer patients after accounting the identified patient‐level factors, Hypothesis 3—PCCM (a high provider communication rating) will be associated with lower AC primary non‐adherence and non‐persistence breast and colon cancer patients after accounting for the identified patient‐level factors.

To achieve these aims, we conducted a retrospective analysis of the treatment experience of a sample of elderly adults (≥65 years old) diagnosed with stage I–III breast cancer (with either nodal involvement or tumor >1 cm) or stage III colon cancer between 2005 and 2013 in the Surveillance, Epidemiology and End Results—Consumer Assessment of Healthcare Providers and Systems Linked Data Resource (SEER‐CAHPS).

## 2 METHODS

### 2.1 Study design and sample

This was a retrospective cohort study using the SEER‐CAHPS database which combines the National Cancer Institute's (NCI) SEER external cancer registry with the Centers for Medicare & Medicaid Services' (CMS) Medicare CAHPS® database. This database includes Medicare enrollment and claims data and survey data evaluating patients' care experiences, including provider communication.^31^ An in‐depth description of the database has been published elsewhere.^32^ Medicare fee‐for‐service enrollees diagnosed (primary) with stage I–III breast cancer (with either nodal involvement or tumor >1 cm) or stage III colon cancer between 2005 and 2013 who had surgery and completed a CAHPS survey within 2 years following their diagnosis were included in the analysis. Patients also had to be aged ≥65 years at diagnosis and have continuous Medicare coverage (Parts A and B) for the year of and after diagnosis. Patients with multiple cancer diagnoses or metastatic disease, enrollment in a health management organization (HMO) 12 months prior to diagnosis or 12 months after diagnosis, or a diagnosis reported via an autopsy or a death certificate were excluded. This study qualified for exemption following a review by the IRB at the study site (#HM20020282).

### 2.2 Study variables

#### 2.2.1 Dependent variables: AC primary non‐adherence and persistence

Adjuvant chemotherapy primary non‐adherence was operationalized as not initiating an AC regimen within 3 months of surgery and was determined by the presence of one or more claims for chemotherapy treatment in the MEDICARE outpatient, inpatient or physician carrier files within 3 months of tumor resection. AC persistence for 3 months and 6 months were determined by assessing whether a patient had chemotherapy claims over a period of 3 or 6 consecutive months, respectively. All outcome variables were defined in terms of their negative, so the modeled outcome was the likelihood of AC primary non‐adherence, non*‐*persistence at 3 months, and non*‐*persistence at 6 months.

#### 2.2.2 Independent variables: PCCM

The Medicare CAHPS survey's composite measure, “Doctor Communication,” was used to assess patients' rating of provider communication during their cancer care experience. It is scored from 0 to 100 and includes the following four items: (1) *Provider explains things to me*: How often did your personal doctor explain things in a way that was easy to understand? (2) *Provider listens to me*: How often did your personal doctor listen carefully to you? (3) *Provider respects me*: How often did your personal doctor show respect for what you had to say? (4) *Provider spends enough time with me*: How often did your personal doctor spend enough time with you? Since we expected there to be ceiling effects in the data as with previous studies,^33^, ^34^ this variable was dichotomized according to two thresholds: 0–89 = no PCCM; 90–100 = PCCM, and 0–99 = no PCCM; 100 = PCCM.

#### 2.2.3 Covariates: Patient‐level factors: Patient characteristics, disease and treatment factors, and patient experience

Patient characteristics including age, sex, race and ethnicity, marital status, education, use of a Spanish survey, assistance with survey (survey proxy), urban/rural dwelling, and Medicaid insurance status were examined. Patients' year of diagnosis and co‐morbidities, and self‐assessments of physical and mental health were used to assess disease and treatment‐related factors. Patient's mental and physical well‐being were evaluated using survey items that ask patients to: “Rate their general health status” and “Rate their mental health” using the following response scale: 1 = “Excellent” 2 = “Very Good” 3 = “Good” 4 = “Fair” 5 = “Poor”. Patient experience was evaluated using the following CAHPS survey items: (1) “Using any number from 0 to 10, where 0 is the worst personal doctor possible and 10 is the best personal doctor possible, what number would you use to rate your personal doctor?” (2) “Using any number from 0 to 10, where 0 is the worst specialist possible and 10 is the best specialist possible, what number would you use to rate that specialist?” and (3) “Using any number from 0 to 10, where 0 is the worst health care possible and 10 is the best health care possible, what number would you use to rate all your health care in the last 6 months?”. These variables were also dichotomized in anticipation of ceiling effects. Scores of 9 or 10 were considered “high quality” care ratings.

### 2.3 Statistical analyses

We conducted descriptive analyses for all study variables. Univariate logistic regressions were used to evaluate associations between PCCM and the identified covariates: patient characteristics, disease and treatment factors, and indicators of the patient experience such as higher patient care ratings and higher general health and mental health status. Univariate logistic regressions were also used to assess relationships between PCCM and non‐adherence to AC guidelines (primary non‐adherence and non‐persistence at 3 and 6 months). Multiple logistic regression was used to determine the factors associated with primary non‐adherence and persistence of AC administered. Variables with a univariable test result of *p* < 0.25 were entered in the multivariable model that was fit using backward stepwise selection, with improvement in Akaike information criterion being the continuation criterion in the stepwise algorithm. Given the collinearity between age and age group, if both variables were significant in univariate models, the continuous age variable was entered into the multivariate model since it conveyed more information. The PCCM thresholds of 90 and 100 were used separately, with the dichotomous PCCM variable with a lower *p*‐value in univariate models entered into the multivariate model. Possible interactions between variables were also evaluated. All analyses were conducted in R Version 3.6.1.

## 3 RESULTS

### 3.1 PCCM and patient‐level factors

Patient‐centered communication and the patient‐level factors examined in this study are summarized in Table 1. Most patients were female (94%), White (87%), non‐Hispanic (95%), and residents of a Big Metro (46%) or Metro (36%) area. The average age was approximately 76 years old. The most common responses for general health status and mental health status were Good (35%) and Excellent (34%), respectively. Though a majority (52%) of patients rated their personal doctor highly, most patients rated their specialist (65%), and healthcare (55%) as low/average. Breast cancer patients were less likely to be diagnosed between 2007 and 2009 (*p* < 0.01). Most patients experienced PCCM as indicated by both PCCM thresholds: PCCM‐90 (73%) and PCCM‐100 (58%). The only statistically significant difference in PCCM that emerged by cancer site was that breast cancer patients were more likely to report that their personal doctor always listened carefully to them (*p* = 0.02).

**TABLE 1:** Patient characteristics, disease and treatment factors, patient experience, and patient‐centered communication (PCCM) by cancer site.

Variable^a^	Level	Overall (*n* = 577)	Breast (*n* = 500)	Colon (*n* = 77)	*p*‐Value
Patient characteristics					
Age		75.89 (6.92)	75.79 (6.87)	76.56 (7.28)	0.365
Sex	Female	543 (94.1)	496 (99.2)	47 (61.0)	<0.001
	Male	34 (5.9)	4 (0.8)	30 (39.0)	
Race	Asian	10 (1.7)	7 (1.4)	3 (3.9)	0.309
	Black	39 (6.8)	33 (6.6)	6 (7.8)	
	Other	26 (4.5)	21 (4.2)	5 (6.5)	
	White	502 (87.0)	439 (87.8)	63 (81.8)	
Hispanic	No	548 (95.0)	475 (95.0)	73 (94.8)	1
	Yes	29 (5.0)	25 (5.0)	4 (5.2)	
Urbanity	Big Metro	266 (46.1)	231 (46.2)	35 (45.5)	0.221
	Less Urban	50 (8.7)	43 (8.6)	7 (9.1)	
	Metro	205 (35.5)	173 (34.6)	32 (41.6)	
	Rural	12 (2.1)	10 (2.0)	2 (2.6)	
	Urban	44 (7.6)	43 (8.6)	1 (1.3)	
Marital	Divorced	54 (9.4)	48 (9.6)	6 (7.8)	0.555
	Married	265 (45.9)	221 (44.2)	44 (57.1)	
	Separated	1 (0.2)	1 (0.2)	0 (0.0)	
	Single	39 (6.8)	34 (6.8)	5 (6.5)	
	Unknown	30 (5.2)	27 (5.4)	3 (3.9)	
	Unmarried/domestic partner	1 (0.2)	1 (0.2)	0 (0.0)	
	Widowed	187 (32.4)	168 (33.6)	19 (24.7)	
Region	East North Central	31 (5.4)	29 (5.8)	2 (2.6)	0.895
	East/West South Central	75 (13.0)	65 (13.0)	10 (13.0)	
	Mountain	40 (6.9)	33 (6.6)	7 (9.1)	
	NE/Middle Atlantic	98 (17.0)	84 (16.8)	14 (18.2)	
	Other	2 (0.3)	2 (0.4)	0 (0.0)	
	Pacific	224 (38.8)	192 (38.4)	32 (41.6)	
	South Atlantic	68 (11.8)	60 (12.0)	8 (10.4)	
	West North Central	39 (6.8)	35 (7.0)	4 (5.2)	
Survey in Spanish	English	573 (99.3)	497 (99.4)	76 (98.7)	1
	Spanish	4 (0.7)	3 (0.6)	1 (1.3)	
Survey proxy	No	568 (98.4)	494 (98.8)	74 (96.1)	0.199
	Yes	9 (1.6)	6 (1.2)	3 (3.9)	
Patient characteristics					
Medicaid	No	474 (95.0)	409 (94.9)	65 (95.6)	1
	Yes	25 (5.0)	22 (5.1)	3 (4.4)	
Disease and treatment factors					
General health status	Excellent	58 (10.1)	54 (10.8)	4 (5.2)	0.126
	Fair	124 (21.5)	100 (20.0)	24 (31.2)	
	Good	201 (34.8)	171 (34.2)	30 (39.0)	
	Missing	27 (4.7)	24 (4.8)	3 (3.9)	
	Poor	18 (3.1)	16 (3.2)	2 (2.6)	
	Very Good	149 (25.8)	135 (27.0)	14 (18.2)	
Mental health status	Excellent	195 (33.8)	175 (35.0)	20 (26.0)	0.627
	Fair	31 (5.4)	27 (5.4)	4 (5.2)	
	Good	133 (23.1)	115 (23.0)	18 (23.4)	
	Missing	30 (5.2)	24 (4.8)	6 (7.8)	
	Poor	6 (1.0)	5 (1.0)	1 (1.3)	
	Very Good	182 (31.5)	154 (30.8)	28 (36.4)	
Comorbidities	Zero	346 (60.0)	305 (61.0)	41 (53.2)	0.373
	One	168 (29.1)	144 (28.8)	24 (31.2)	
	Two	48 (8.3)	38 (7.6)	10 (13.0)	
	Three	15 (2.6)	13 (2.6)	2 (2.6)	
Diagnosis year	2007–2009	148 (25.6)	117 (23.4)	31 (40.3)	0.006
	2010–2012	331 (57.4)	297 (59.4)	34 (44.2)	
	2013–2015	98 (17.0)	86 (17.2)	12 (15.6)	
Patient experience					
Personal doctor rating	High	301 (52.2)	260 (52.0)	41 (53.2)	0.935
	Low/average	276 (47.8)	240 (48.0)	36 (46.8)	
Specialist rating	High	204 (35.4)	171 (34.2)	33 (42.9)	0.177
	Low/average	373 (64.6)	329 (65.8)	44 (57.1)	
Healthcare rating	High	258 (44.7)	224 (44.8)	34 (44.2)	1
	Low/average	319 (55.3)	276 (55.2)	43 (55.8)	
PCCM					
PCCM 90	No PCCM	112 (27.3)	90 (25.6)	22 (37.3)	0.089
	PCCM	298 (72.7)	261 (74.4)	37 (62.7)	
PCCM 100	No PCCM	174 (42.4)	141 (40.2)	33 (55.9)	0.511
	PCCM	236 (57.6)	210 (59.8)	26 (44.1)	
PCCM					
Doctor explains things to me	Always	312 (54.1)	271 (54.2)	41 (53.2)	0.756
	Usually	81 (14.0)	67 (13.4)	14 (18.2)	
	Sometimes	12 (2.1)	10 (2.0)	2 (2.6)	
	Never	2 (0.3)	2 (0.4)	0 (0.0)	
	Missing	170 (29.5)	150 (30.0)	20 (26.0)	
Doctor listens to me	Always	312 (54.1)	272 (54.4)	40 (51.9)	0.024
	Usually	79 (13.7)	66 (13.2)	13 (16.9)	
	Sometimes	15 (2.6)	9 (1.8)	6 (7.8)	
	Never	1 (0.2)	1 (0.2)	0 (0.0)	
	Missing	170 (29.5)	152 (30.4)	18 (23.4)	
Doctor respects me	Always	332 (57.5)	287 (57.4)	45 (58.4)	0.464
	Usually	60 (10.4)	48 (9.6)	12 (15.6)	
	Sometimes	14 (2.4)	12 (2.4)	2 (2.6)	
	Never	1 (0.2)	1 (0.2)	0 (0.0)	
	Missing	170 (29.5)	152 (30.4)	18 (23.4)	
Doctor spends enough time with me	Always	273 (47.3)	238 (47.6)	35 (45.5)	0.431
	Usually	109 (18.9)	89 (17.8)	20 (26.0)	
	Sometimes	17 (2.9)	15 (3.0)	2 (2.6)	
	Never	4 (0.7)	3 (0.6)	1 (1.3)	
	Missing	174 (30.2)	155 (31.0)	19 (24.7)	

^a^ Continuous variable summarized with mean (SD); all categorical variables summarized with count (frequency).

### 3.2 Hypothesis 1: Rates of non‐adherence to AC guidelines

Each AC non‐adherence outcome differed significantly by cancer type (see Table 2). About 69% of breast cancer patients did not initiate chemotherapy compared to 43% of colon cancer patients (*p* < 0.001). About 81% of breast cancer patients did not persist in their AC for 3 months compared to 46% of colon cancer patients (*p* < 0.001). Finally, 89% of breast cancer patients did not persist their AC for 6 months compared to 62% of colon cancer patients (*p* < 0.001).

**TABLE 2:** Non‐adherence to adjuvant chemotherapy (AC) guidelines by cancer site.

Variable^a^	Level	Overall (*n* = 577)	Breast (*n* = 500)	Colon (*n* = 77)	*p*‐Value
AC non‐adherence					
Primary AC non‐adherence	False	198 (34.3)	154 (30.8)	44 (57.1)	<0.001
	True	379 (65.7)	346 (69.2)	33 (42.9)	
AC non‐persistence at 3 months of chemotherapy	False	136 (23.6)	94 (18.8)	42 (54.5)	<0.001
	True	441 (76.4)	406 (81.2)	35 (45.5)	
AC non‐persistence at 6 months	False	84 (14.6)	55 (11.0)	29 (37.7)	<0.001
	True	493 (85.4)	445 (89.0)	48 (62.3)	

^a^ Continuous variable summarized with mean (SD); all categorical variables summarized with count (frequency).

### 3.3 Hypothesis 2: Correlates of PCCM

#### 3.3.1 PCCM‐100

Several demographic, disease, treatment, and patient experience factors were significantly associated with PCCM‐100 (see Table 3). Male patients (*p* = 0.04) and those who rated rating their personal doctor (*p* < 0.001), specialist (*p* = 0.003), and healthcare generally (*p* < 0.001) as low/average were less likely to report PCCM‐100 than female patients, and those who reported a high rating for their personal doctor, specialist, and healthcare, respectively. There were no significant univariate associations between any of the AC non‐adherence outcomes and reporting PCCM‐100.

**TABLE 3:** Odds ratios for the associations between patient‐centered communication (PCCM) and patient demographic, disease, treatment, and experience factors.

Variable	Level	PCCM‐100		PCCM‐90	
		OR (95% CI)	*p*‐Value	OR (95% CI)	*p*‐Value
Age	(Numeric)	0.98 (0.95, 1.01)	0.18	1.00 (0.97, 1.03)	0.82
Sex	Female	REF	0.04	REF	
	Male	0.42 (0.17, 0.97)		0.61 (0.26, 1.48)	0.25
Race	Asian	REF	0.76	REF	
	Black	2.40 (0.44, 14.35)		0.84 (0.11, 4.81)	0.86
	Other	1.67 (0.29, 10.66)		0.80 (0.09, 5.07)	
	White	1.80 (0.39, 9.24)		1.10 (0.16, 5.21)	
Hispanic	No	REF	0.36	REF	
	Yes	0.65 (0.25, 1.65)		0.50 (0.20, 1.32)	0.14
Urbanity	Big Metro	REF	0.12	REF	
	Less Urban	2.39 (1.03, 6.26)		1.91 (0.75, 5.88)	
	Metro	0.75 (0.49, 1.15)		0.87 (0.54, 1.41)	
	Rural	1.16 (0.28, 5.80)		0.61 (0.15, 3.07)	
	Urban	0.86 (0.39, 1.91)		0.97 (0.42, 2.44)	0.60
Marital	Divorced	REF	0.67	REF	
	Married	1.15 (0.58, 2.25)		0.99 (0.46, 2.04)	
	Single	1.40 (0.53, 3.86)		0.95 (0.33, 2.82)	
	Unknown	1.98 (0.62, 7.15)		3.00 (0.70, 20.89)	
	Widowed	1.01 (0.50, 2.03)		1.09 (0.49, 2.31)	0.79
Region	East North Central	REF	0.09	REF	
	East/West South Central	1.18 (0.38, 3.47)		2.07 (0.59, 7.14)	
	Mountain	0.27 (0.08, 0.83)		0.67 (0.19, 2.20)	
	NE/Middle Atlantic	0.58 (0.20, 1.55)		0.82 (0.27, 2.30)	
	Pacific	0.52 (0.19, 1.29)		0.86 (0.30, 2.22)	
	South Atlantic	0.69 (0.23, 1.95)		1.09 (0.33, 3.32)	
	West North Central	0.48 (0.14, 1.63)		0.71 (0.19, 2.60)	0.53
Survey in Spanish	English	REF	0.75	REF	
	Spanish	1.48 (0.14, 31.97)		0.75 (0.07, 16.23)	0.82
Survey proxy	No	REF	0.12	REF	
	Yes	0.29 (0.04, 1.36)		0.14 (0.02, 0.68)	0.01
Medicaid	No	REF	0.22	REF	
	Yes	0.59 (0.24, 1.39)		0.53 (0.22, 1.32)	0.15
General health status	Excellent	REF	0.17	REF	
	Fair	0.52 (0.22, 1.17)		0.56 (0.20, 1.36)	
	Good	0.67 (0.29, 1.45)		0.75 (0.28, 1.79)	
	Missing	1.33 (0.31, 7.00)		0.72 (0.16, 3.92)	
	Poor	0.28 (0.07, 0.99)		0.67 (0.16, 3.03)	
	Very Good	0.84 (0.36, 1.90)		0.79 (0.29, 1.95)	0.81
Mental health status	Excellent	REF	0.49	REF	
	Fair	0.52 (0.22, 1.22)		0.54 (0.22, 1.37)	
	Good	0.62 (0.36, 1.05)		0.64 (0.36, 1.15)	
	Missing	0.72 (0.25, 2.12)		0.66 (0.22, 2.23)	
	Poor	0.56 (0.07, 4.77)		0.30 (0.03, 2.59)	
	Very Good	0.73 (0.45, 1.19)		0.89 (0.50, 1.55)	0.47
Comorbidities	(Numeric)	0.90 (0.70, 1.15)	0.39	0.88 (0.68, 1.16)	0.36
Diagnosis year	2007–2009	REF	0.11	REF	
	2010–2012	1.28 (0.82, 1.99)		1.35 (0.83, 2.19)	
	2013–2015	2.10 (1.06, 4.28)		1.79 (0.85, 4.02)	0.27
Personal doctor rating	High	REF	<0.001	REF	
	Low/average	0.06 (0.04, 0.11)		0.05 (0.03, 0.09)	<0.001
Specialist rating	High	REF	0.003	REF	
	Low/average	0.55 (0.37, 0.82)		0.47 (0.29, 0.75)	0.001
Healthcare rating	High	REF	<0.001	REF	
	Low/average	0.16 (0.10, 0.24)		0.17 (0.10, 0.27)	<0.001
Primary adjuvant chemotherapy (AC) non‐adherence	False	REF	0.42	REF	
	True	1.19 (0.79, 1.78)		1.66 (1.06, 2.59)	0.02
AC non‐persistence at 3 months of chemotherapy	False	REF	0.16	REF	
	True	1.39 (0.88, 2.18)		1.60 (0.98, 2.58)	0.06
AC non‐persistence at 6 months	False	REF	0.35	REF	
	True	1.29 (0.75, 2.10)		1.45 (0.82, 2.53)	0.20

#### 3.3.2 PCCM‐90

Several factors were significantly associated with the more inclusive definition of PCCM (PCCM‐90). Those using a proxy to complete the survey were significantly less likely to report PCCM‐90 (*p* = 0.01). Similar to above, those rating their personal doctor (*p* < 0.001), specialist (*p* = 0.001), and healthcare (*p* < 0.001) as low/average were less likely to report PCCM‐90. Finally, those reporting PCCM‐90 were more likely to not initiate their AC (*p* = 0.02) and persist their AC for 3 months (*p* = 0.057), although the latter finding was marginally significant.

### 3.4 Hypothesis 3: Patient‐level correlates at three levels of non‐adherence to AC guidelines

#### 3.4.1 Primary non‐adherence to AC

Several variables exhibited significant univariate associations with primary non‐adherence to AC (see Table 4). Those variables that were entered into the multivariate model were age, sex, marital status, number of comorbidities, diagnosis year group, cancer type, specialist rating, and PCCM‐100. Backward selection yielded a multivariate model with three variables associated with the likelihood of not initiating AC. Specifically, increasing age (*p* < 0.001) and diagnosis year group later than 2007–2009 (2010–2012 OR = 2.51, 95% CI (1.53, 4.15), 2013–2015 OR = 2.00, 95% CI (0.97, 4.21), *p* = 0.001) were significantly associated with primary non‐adherence, and those with colon cancer (*p* < 0.001) were significantly less likely to exhibit primary nonadherence.

**TABLE 4:** Odds ratios for the associations between patient‐level factors and adjuvant chemotherapy (AC) outcomes.

Variable	Level	AC non‐initiation				AC non‐persistence at 3 months				AC non‐persistence at 6 months			
		Univariate		Multivariate		Univariate		Multivariate		Univariate		Multivariate	
		OR (95% CI)	*p*‐Value	OR (95% CI)	*p*‐Value	OR (95% CI)	*p*‐Value	OR (95% CI)	*p*‐Value	OR (95% CI)	*p*‐Value	OR (95% CI)	*p*‐Value
Age	(Numeric)	1.09 (1.06, 1.12)	<0.001	1.10 (1.06, 1.14)	<0.001	1.07 (1.04, 1.11)	<0.001	1.10 (1.06, 1.15)	<0.001	1.05 (1.01, 1.09)	0.01	1.07 (1.03, 1.13)	0.01
Sex	Female	REF	0.02			REF	<0.001			REF	<0.001		
	Male	0.44 (0.22, 0.89)				0.32 (0.16, 0.65)				0.28 (0.13, 0.61)			
Race	Asian	REF	0.27			REF	0.52			REF	0.18		
	Black	0.45 (0.09, 1.88)				0.50 (0.07, 2.36)				0.32 (0.02, 2.05)			
	Other	0.96 (0.17, 4.54)				0.83 (0.11, 4.58)				0.47 (0.02, 3.47)			
	White	0.85 (0.18, 3.11)				0.84 (0.13, 3.41)				0.71 (0.03, 3.86)			
Hispanic	No	REF	0.41			REF	0.94			REF	0.91		
	Yes	0.73 (0.34, 1.59)				0.97 (0.42, 2.49)				1.07 (0.40, 3.70)			
Urbanity	Big Metro	REF	0.3			REF	0.46			REF	0.99		
	Less Urban	0.75 (0.41, 1.42)				0.80 (0.41, 1.62)				1.09 (0.48, 2.79)			
	Metro	0.91 (0.62, 1.33)				0.99 (0.65, 1.52)				1.07 (0.64, 1.81)			
	Rural	0.50 (0.15, 1.65)				0.62 (0.19, 2.39)				0.89 (0.22, 5.90)			
	Urban	1.71 (0.84, 3.79)				1.97 (0.85, 5.36)				1.12 (0.47, 3.10)			
Marital	Divorced	REF	0.01			REF	0.08			REF	0.06		
	Married	0.81 (0.43, 1.48)				0.94 (0.47, 1.79)				0.92 (0.40, 1.94)			
	Single	0.97 (0.41, 2.31)				0.89 (0.35, 2.28)				1.75 (0.52, 6.89)			
	Unknown	0.81 (0.32, 2.07)				1.15 (0.41, 3.41)				1.30 (0.38, 5.18)			
	Widowed	1.71 (0.88, 3.27)				1.76 (0.84, 3.57)				1.77 (7.20, 4.08)			
Region	East North Central	REF	0.9			REF	0.3			REF	0.29		
	East/West South Central	0.82 (0.32, 2.00)				1.08 (0.37, 2.87)				2.21 (0.59, 7.96)			
	Mountain	0.76 (0.27, 2.07)				0.68 (0.22, 1.97)				0.91 (0.24, 3.17)			
	NE/Middle Atlantic	0.70 (0.28, 1.65)				0.69 (0.25, 1.72)				0.85 (0.26, 2.39)			
	Pacific	0.86, 0.36, 1.92)				1.19 (0.45, 2.83)				1.29 (0.41, 3.40)			
	South Atlantic	0.58 (0.23, 1.43)				0.65 (0.23, 1.70)				0.68 (0.20, 1.97)			
	West North Central	0.82 (0.29, 2.26)				1.33 (0.41, 4.39)				1.69 (0.41, 7.39)			
Survey in Spanish	English	*				REF	0.21			*			
	Spanish					0.31 (0.04, 2.56)							
Survey proxy	No	REF	0.95			REF	0.49			REF	0.11		
	Yes	1.05 (0.27, 5.00)				0.61 (0.16, 2.93)				0.33 (0.09, 1.60)			
Medicaid	No	REF	0.73			REF	0.57			REF	0.28		
	Yes	1.17 (0.51, 2.91)				1.34 (0.53, 4.10)				2.20 (0.63, 13.86)			
General health status	Excellent	REF	0.57			REF	0.69			REF	0.17		
	Fair	0.87 (0.45, 1.65)				0.55 (0.24, 1.18)				0.44 (0.14, 1.14)			
	Good	1.24 (0.67, 2.28)				0.65 (0.29, 1.33)				0.46 (0.15, 1.15)			
	Missing	1.35 (0.51, 3.75)				0.60 (0.20, 1.84)				0.33 (0.09, 1.21)			
	Very Good	1.03 (0.54, 1.92)				0.73 (0.32, 1.56)				0.78 (0.25, 2.12)			
Mental health status	Excellent	REF	0.4			REF	0.22			REF	0.04		
	Fair	0.99 (0.46, 2.21)				1.56 (0.64, 4.37)				1.33 (0.48, 4.71)			
	Good	1.62 (1.01, 2.63)				1.61 (0.95, 2.79)				1.54 (0.81, 3.05)			
	Missing	1.08 (0.49, 2.46)				0.65 (0.29, 1.48)				0.39 (0.17, 0.95)			
	Very Good	1.27 (0.83, 1.94)				1.37 (0.85, 2.21)				1.36 (0.76, 2.44)			
Region × GHS	(Many levels)		0.52				0.83				*		
Region × age	(Many levels)		0.62				0.72				0.97		
Comorbidities	(Numeric)	1.19 (0.95, 1.53)	0.13			1.31 (1.01, 1.75)	0.05	1.53 (1.08, 2.22)	0.02	1.24 (0.90, 1.75)	0.21		
Diagnosis year	2007–2009	REF	<0.001	REF		REF	<0.001	REF		REF	<0.001	REF	
	2010–2012	2.35 (1.58, 3.52)		2.51 (1.53, 4.15)		2.22 (1.44, 3.42)		2.04 (1.17, 3.54)		2.82 (1.69, 4.71)		2.34 (1.22, 4.49)	
	2013–2015	2.05 (1.21, 3.52)		2.00 (0.97, 4.21)	0.001	2.25 (1.25, 4.20)		1.78 (0.79, 4.23)	0.04	2.39 (1.21, 5.03)		1.76 (0.71, 4.77)	0.04
Cancer	Breast	REF		REF		REF		REF		REF		REF	
	Colon	0.33 (0.20, 0.54)	<0.001	0.31 (0.17, 0.56)	<0.001	0.19 (0.12, 0.32)	<0.001	0.17 (0.09, 0.32)	<0.001	0.20 (0.12, 0.35)	<0.001	0.23 (0.11, 0.45)	<0.001
Personal doctor rating	High	REF	0.45			REF	0.7			REF	0.85		
	Low/average	0.88 (0.62, 1.24)				0.93 (0.63, 1.36)				0.96 (0.60, 1.52)			
Specialist rating	High	REF	0.1			REF	0.23			REF	0.57		
	Low/average	1.35 (0.94, 1.92)				1.28 (0.86, 1.89)				1.15 (0.71, 1.84)			
Healthcare rating	High	REF	0.66			REF	0.97			REF	0.73		
	Low/average	1.08 (0.76, 1.52)				1.01 (0.68, 1.48)				1.08 (0.68, 1.72)			
PCCM 90	No	REF	0.42			REF	0.06	REF		REF	0.2		
	Yes	1.19 (0.79, 1.78)				1.60 (0.98, 2.58)		1.61 (0.93, 2.76)	0.13	1.45 (0.81, 2.53)			
PCCM 100	No	REF	0.02			REF	0.16			REF	0.35		
	Yes	1.66 (1.06, 2.59)				1.39 (0.88, 2.18)				1.29 (0.75, 2.19)			

* Complete separation of the data or model did not converge, did not add to multivariate model.

#### 3.4.2 Non‐persistence to AC guidelines at 3 months

Several variables, identified through univariate analysis, exhibited significant associations with AC non‐persistence at 3 months. Age, sex, marital status, Spanish version of the survey, mental health status, number of comorbidities, diagnosis year group, cancer type, specialist rating, and PCCM‐90 were entered into the multivariate model. Backward selection yielded a multivariate model with five variables associated with the likelihood of AC non‐persistence at 3 months. Specifically, increasing age (*p* < 0.001), greater number of comorbidities (*p* = 0.02), diagnosis group later than 2007–2009 (2010–2012 OR = 2.04, 95% CI (1.17, 3.54), 2013–2015 OR = 1.78, 95% CI (0.79, 4.23), *p* = 0.04), and PCCM‐90 (*p* = 0.13) were associated with higher rates of non‐persistence at 3 months, and patients with colon cancer (*p* < 0.001) were significantly less likely to exhibit non‐persistence at 3 months.

#### 3.4.3 Non‐persistence to AC guidelines at 6 months

Several variables exhibited significant associations with non‐persistence of AC at 6 months. Age, sex, race, marital status, using a proxy for the survey, general health status, mental health status, number of comorbidities, diagnosis year group, cancer type, and PCCM‐90 were entered into the multivariate model. Backward selection yielded a multivariate model with three variables associated with the likelihood of non‐persistence of chemotherapy at 6 months. Specifically, increasing age (*p* = 0.01) and diagnosis year group later than 2007–2009 (2010–2012 OR = 2.34, 95% CI (1.22, 4.49), 2013–2015 OR = 1.76, 95% CI (0.71, 4.77), *p* = 0.04) were associated with non‐persistence at 6 months, and colon cancer (*p* < 0.001) was significantly associated with higher non‐persistence at 6 months compared to breast cancer.

## 4 DISCUSSION AND CONCLUSION

### 4.1 Discussion

Despite the known benefits of AC in eligible breast and colon cancer patients, evidence has demonstrated that significant proportions of these patients are non‐adherent to recommended treatment protocols. To illuminate this concerning lack of adherence, we explored three levels of non‐adherence (primary non‐adherence, non‐persistence at 3 months, and non‐persistence at 6 months) to AC guidelines among a nationally representative sample of breast or colon cancer patients. We examined the relationship between patient characteristics, disease and treatment factors, and patient experience including ratings of physician communication and their respective associations with the three levels of non‐adherence. We found higher rates of AC non‐adherence among older patients, breast cancer patients and those diagnosed after the 2007–2009 time period, compared to younger patients, colon cancer patients and those diagnosed between 2007 and 2009. Our findings confirmed that AC non‐adherence continues to be a problem in the study population and estimated the influence of age and disease and treatment factors on these outcomes. The role of PCCM in AC guideline non‐adherence is not clear; however, as it appears to be affected by the stage of care and level of PCCM.

#### 4.1.1 Rates of non‐adherence to AC guidelines

The rates of non‐adherence that we obtained for breast and colon cancer patients were consistent with the upper ranges of previously published rates found in breast cancer patients.^9^, ^10^, ^11^, ^12^ The high rates in our study may be attributed to (a) the older age and type of insurance held by our patient sample with older and uninsured patients being less adherent than younger well insured patients and (b) varying definitions of non‐adherence across studies.

#### 4.1.2 Variability in PCCM associations with gender and AC non‐adherence over time

The results of our univariate analyses, such as PCCM's associations with patient gender,^35^ personal doctor ratings,^36^, ^37^ specialist type,^38^ and health care ratings^35^, ^39^ aligned with the findings of other studies and confirmed the variability of these relationships. This variability is particularly true in the relationship between PCCM and gender, with some studies reporting no differences in PCCM by gender and others reporting that women were more likely than men to report PCCM^35^, ^40^, ^41^ Furthermore, our result that only one PCCM construct, PCCM‐90 was correlated with non‐persistence at 3 months was counterintuitive, particularly as higher patient scores on PCCM‐90 were associated with lower adherence. It was expected that optimal communication would be associated with lower non‐adherence, but the opposite was true in this case. More information is needed to understand whether communication supports meeting treatment recommendations, patient goals, both, or neither. Additionally, comorbidities and PCCM‐90 were associated with non‐persistence at 3 months but not the other two levels of AC non‐adherence. This highlights the emergence of comorbidities and their discussion after beginning AC as a potentially critical aspect of the relationship between PCCM and AC non‐adherence.

#### 4.1.3 Race/ethnicity and AC non‐adherence

The correlations we identified between older age,^12^, ^16^, ^17^ a breast cancer diagnosis,^10^ and a post‐2009 cancer diagnosis^42^ and AC non‐adherence were consistent with previous research. Alternatively, unlike other studies,^11^, ^12^, ^16^, ^19^, ^20^ we did not find a correlation between AC non‐adherence and race/ethnicity. Limited representation of racial‐ethnic minorities in the sample may have inhibited our ability to detect relationship this relationship that is likely influenced by other factors such as income and insurance status.

#### 4.1.4 Limitations

While the linked SEER‐CAHPS database is large and nationally representative, our sample size was significantly limited by the number of patients who completed a CAHPS within a time frame such that one could reasonably assume that their responses applied to their cancer care experience. Thus, the sample sizes of colon cancer patients and underrepresented racial and ethnic groups were small. Furthermore, the type of chemotherapy agents used chemotherapy were not always specified in the dataset, prohibiting us from determining whether patients completed the entire regimen as recommended by the NCCN guidelines. Study strengths included comparing the rates of AC non‐adherence between two large cancer patient populations, focusing on patient‐level factors to inform patient‐centered approaches to decreasing AC non‐adherence, and the use of multiple levels of PCCM, and AC non‐adherence to elucidate more nuanced trends in the relationship between these factors in oncology practice.

#### 4.1.5 Practice implications

Our study findings suggest that PCCM may be operating in two directions depending on other factors such as patients' comorbidities or symptom experience and providers' responses to these issues. Assessing both value‐concordant treatment and AC guideline non‐adherence, particularly when comorbidities are present, could support changes in practice to achieve patient‐desired outcomes. Data collection methods should be modified to systematically obtain data on AC non‐adherence and its potential causes across healthcare organizations to enable researchers and practitioners to more accurately assess and address this important issue in oncology.

### 4.2 Conclusions

A large proportion of elderly breast and colon cancer patients in the U.S. are not receiving AC as recommended. Variations in disease and treatment experiences are likely contributors to differences in non‐adherence rates to AC guidelines by cancer diagnosis and type of non‐adherence. The relationship between PCCM and AC non‐adherence differed by level of PCCM (PCCM‐90) and non‐adherence (non‐persistence at 3 months), warranting the use of measures that can effectively capture differences in these constructs. Higher survey participation rates, particularly among racial and ethnic minorities, would bolster efforts to understand their communication and AC experiences to improve clinical and patient‐desired outcomes in these patient populations.

## AUTHOR CONTRIBUTIONS

**Kerri‐Anne R. Mitchell:** Conceptualization (lead); data curation (lead); funding acquisition (equal); investigation (lead); project administration (lead); writing – original draft (lead); writing – review and editing (lead). **Joseph R. Boyle:** Data curation (equal); formal analysis (lead); methodology (equal); software (lead); writing – original draft (supporting); writing – review and editing (supporting). **Lenka Juricekova:** Data curation (supporting); writing – review and editing (supporting). **Richard F. Brown:** Conceptualization (supporting); supervision (lead); writing – review and editing (supporting).

## FUNDING INFORMATION

This study was supported by the National Cancer Institute's T32 Postdoctoral Training Program (Grant No. 2T32CA093423).

## CONFLICT OF INTEREST STATEMENT

The authors do not have any conflicts of interest to declare.

## Data Availability

The data that support the findings of this study were obtained from the SEER‐CAHPS Data Resource and requires a data use agreement with the National Cancer Institute.
